# Comparing distributions of polygenic risk scores of type 2 diabetes and coronary heart disease within different populations

**DOI:** 10.1371/journal.pone.0179238

**Published:** 2017-07-05

**Authors:** Sulev Reisberg, Tatjana Iljasenko, Kristi Läll, Krista Fischer, Jaak Vilo

**Affiliations:** 1 University of Tartu, Institute of Computer Science, Tartu, Estonia; 2 Software Technology and Applications Competence Centre, Tartu, Estonia; 3 Quretec Ltd, Tartu, Estonia; 4 University of Tartu, Institute of Mathematics and Statistics, Tartu, Estonia; 5 Estonian Genome Centre, University of Tartu, Tartu, Estonia; Estonian Biocentre, ESTONIA

## Abstract

Polygenic risk scores are gaining more and more attention for estimating genetic risks for liabilities, especially for noncommunicable diseases. They are now calculated using thousands of DNA markers. In this paper, we compare the score distributions of two previously published very large risk score models within different populations. We show that the risk score model together with its risk stratification thresholds, built upon the data of one population, cannot be applied to another population without taking into account the target population’s structure. We also show that if an individual is classified to the wrong population, his/her disease risk can be systematically incorrectly estimated.

## Introduction

Noncommunicable diseases, also known as chronic diseases, are currently responsible for more deaths than all other causes together [1]. Cardiovascular diseases (CVD), cancers, chronic respiratory diseases, and diabetes in particular are responsible for the majority of them [1]. The major cause of death among all CVDs is coronary heart disease (CHD) [1].

It is recognised that CVDs and the type 2 diabetes (T2D) are potentially preventable [1, 2]. For this reason, the early identification of individuals with a high risk of these diseases is most important.

The cause of these diseases is considered to be complex, combining both genetic and environmental factors [1–4]. While environmental factors have been thoroughly studied, it has been a challenge to find the exact most important genetic markers that explain the occurrence of such complex diseases. It is believed and supported by large-scale GWAS studies that genetic risk depends on a large number of genetic markers, each one of them having relatively small effect if taken separately [5].

For this reason, the polygenic risk score (PRS), the risk metric calculated on several single nucleotide polymorphisms (SNP), weighted by their effect-size estimates (logistic/linear regression coefficients from GWAS meta-analysis), can be seen as an approximation of the total genetic risk and is in the focus of the current research. Starting with a few dozens of markers [3, 6], PRSs are now being calculated using hundreds, thousands [7] and even tens of thousands [8] of SNPs.

Several authors have provided PRS models for indicating low and high risks for different diseases or traits–e.g. CHD [8], T2D [7], schizophrenia [9], psychiatric disorders [10], but also for predicting socioeconomic status [11].

Genetic risk estimation is mostly based on percentiles of the PRS distribution in the study cohort [12] and many studies in this field have estimated the relative or absolute risk differences between highest and lowest deciles or quintiles. However, the long-term purpose is to incorporate PRSs in the clinical risk stratification algorithms, to assess the risk levels of individuals outside the original study cohorts. For that reason, absolute thresholds are needed.

It is shown that PRSs, particularly those that consist of up to hundreds of SNPs, are dependent on the discovery cohort [13]. Usually, the selection of SNPs and their corresponding weights are based on previously published meta-analysis, conducted mainly in European-ancestry populations [14, 15] that makes the PRS to be biased towards Europeans [13]. However, to the best of our knowledge it has not been investigated whether the risk estimates that are based on PRS distribution in one cohort are accurate for individuals that do not belong to the cohort.

In this study, we have used two previously published PRSs, both based on thousands of SNPs and compared their distributions within different populations. The populations of interest include Estonia, Europe, America, South-Asia, East-Asia and Africa.

## Materials and methods

PRS is calculated as a sum of weighted effect alleles. The general mathematical formula of the PRS is written as follows:

$$PRS=\sum_{i=1}^{n}w_{i}\cdot X_{i}$$

where X_i_ denotes the effect allele count and w_i_ the weight of the i-th SNP for a certain outcome, accordingly. The number of SNPs included in PRS (denoted with n) varies, depending on the trait/disease.

We used PRS calculation pipelines from two recently published articles. The first is PRS for predicting the risk of CHD (PRS_CHD_), based on 49310 SNPs [8]. It is built on European populations–particularly on Finnish, Dutch and other Western and Southern European ancestries. The second PRS is also built on samples of European descent for predicting T2D (PRS_T2D_), based on 7502 SNPs [7]. In both articles, the effect sizes (ß) for SNPs are estimated in the meta-analyses which were performed using additive models. For PRS_CHD_, weights w_i_ are taken to be equal to the estimated effect sizes ß_i_. In T2D model, an additional parameter π_i_ is used for each SNP to determine the weight, so that *w*_*i*_ = *β*_*i*_ ⋅ *π*_*i*_. That kind of double-weighting of SNPs helps to minimise the bias arising from the “winner’s curse”. For a better comparison, both PRS are scaled over all samples.

In the CHD model, we omitted palindromic SNPs and the calculation was based on 46648 SNPs. According to the supplementary materials of Abraham et al. [8], this does not affect the performance significantly. In addition, we left out 107 SNPs from T2D and 652 SNPs from CHD calculation, due to the missing data (see below). As a result, our calculations were conducted on 7395 and 45996 SNPs accordingly, sharing 5164 common SNPs by ID. To make sure that omitted SNPs have negligible effect on the results, we fitted logistic regression model for prevalent type 2 diabetes, including only PRS_T2D_ as a covariate (there were 1199 common samples with Läll et al. [7]). The odds ratios (OR) remain similar–our OR is 1.76 (95% confidence interval 1.26..2.46) compared to 1.61 (1.16..2.24) in the original article.

In order to calculate the PRS_CHD_ and PRS_T2D_ in different populations, we used 1000 Genomes Project data from Phase 3 (October 2014) release [16]. It contains samples of 2504 individuals from 5 super-populations (in this paper called populations): East-Asia (EAS, 504 individuals), South-Asia (SAS, 489), Europe (EUR, 503), America (AMR, 347) and Africa (AMR, 661). In addition, to represent the Estonian population (EST), we added 2244 samples having a full DNA sequence available, from the Estonian Biobank [17]–a population-based biobank, holding samples of approximately 5% of the Estonian adult population [17]. SNPs were extracted from both datasets either by their ID that was mentioned in the PRS model or by their alias, found from dbSNP [18]. No imputation was performed. SNPs that were not present in VCF, are listed in supplementary materials (S1 File) and were left out from the analysis.

PRSs were calculated by using PLINK (v1.9) [19]. Output files are available in S2 File.

For the genetic risk estimation, individuals are divided into quintiles, based on the PRS values in each study cohort. In both models the risk is considered to be highest for individuals in the top PRS quintile and lowest for the bottom PRS quintile.

Finally, the distributions of the scores in all populations were plotted, quintiles calculated and compared by using R version 3.2.3.

As there is no phenotype data in 1000 Genome Project, we were unable to analyse the association between disease prevalence and the risk score within different populations.

In order to explore the genetic variability of the input data between populations and to better interpret the outcomes, we also performed a Principal Component Analysis (PCA) of SNPs.

## Results

**Fig 1:**
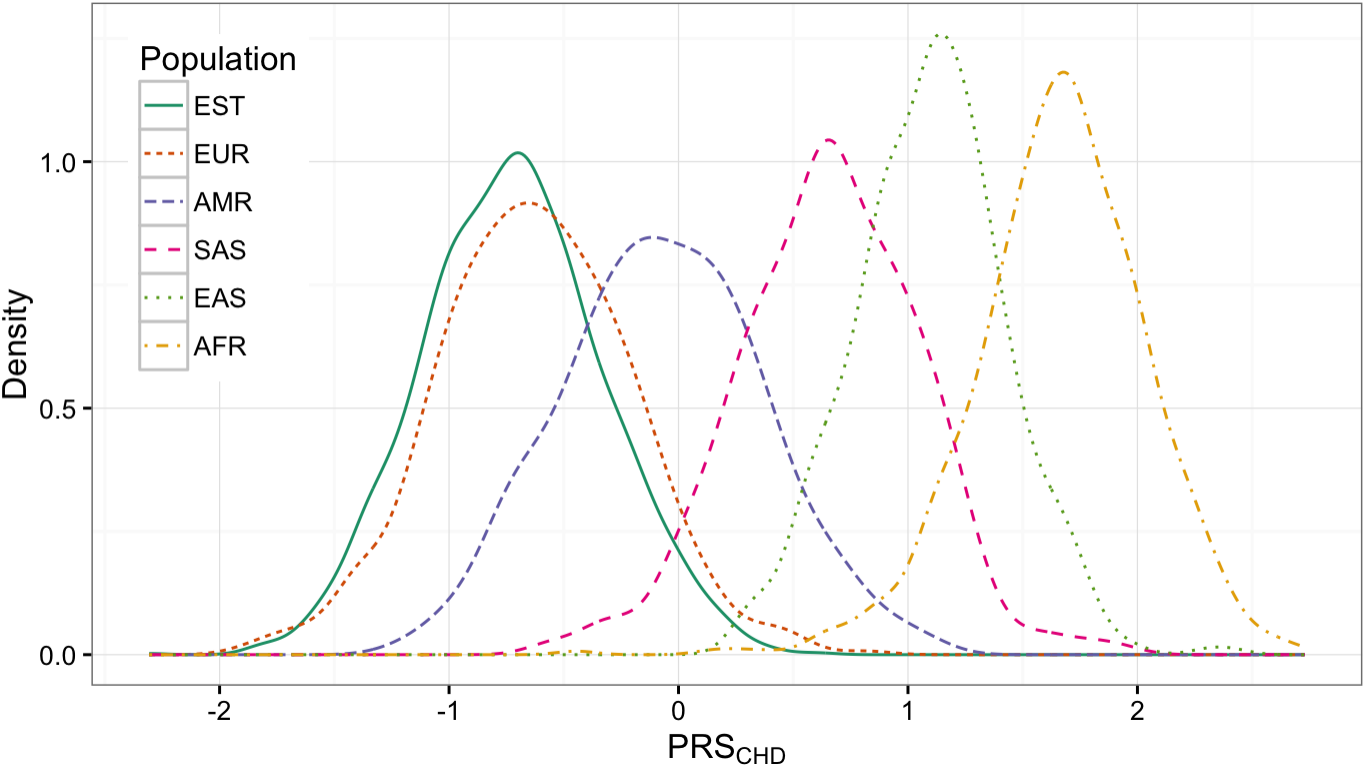
PRS_CHD_ distributions in different populations.

**Fig 2:**
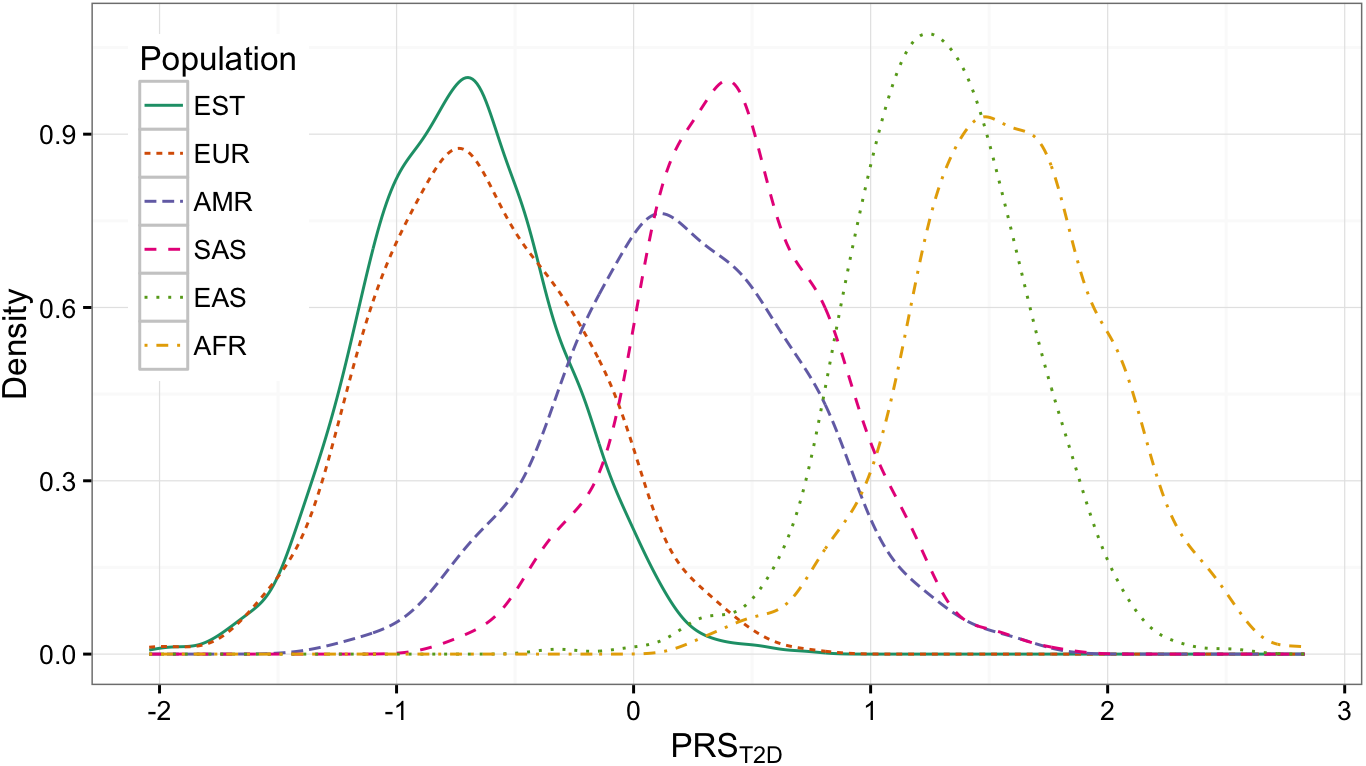
PRS_T2D_ distributions in different populations.

The observed distributions of PRS_CHD_ and PRS_T2D_ are shown in Fig 1 and Fig 2 accordingly. The corresponding quintiles are given in Table 1.

**Table 1:** PRS_CHD_ and PRS_T2D_ distribution means, mins, maxs and quintiles (20%, 40%, 60%, 80%) of SNPs in the model in different populations.

PRS model	Popu-lation	Mean PRS with 95% confidence intervals	PRS quintiles						Correlation between PRS and first component of PCA (with p-value)
			Min	20%	40%	60%	80%	Max	
**CHD**	**EST**	-0.73 (-0.74..-0.71)	-2.31	-1.06	-0.83	-0.63	-0.4	0.63	-0.05 (1.2·10^−2^)
	**EUR**	-0.63 (-0.67..-0.59)	-1.89	-0.97	-0.74	-0.52	-0.28	0.82	0.19 (13·10^−5^)
	**AMR**	-0.07 (-0.12..-0.03)	-1.22	-0.45	-0.18	0.06	0.30	1.11	-0.40 (7.8·10^−15^)
	**SAS**	0.65 (0.62..0.69)	-0.60	0.32	0.58	0.75	0.98	1.95	-0.19 (2.0·10^−5^)
	**EAS**	1.10 (1.07..1.13)	0.25	0.84	1.02	1.18	1.35	2.41	-0.01 (7.5·10^−1^)
	**AFR**	1.66 (1.63..1.69)	-0.45	1.39	1.60	1.76	1.96	2.73	-0.50 (1.5·10^−42^)
**T2D**	**EST**	-0.73 (-0.74..-0.71)	-2.04	-1.07	-0.83	-0.63	-0.40	0.72	0.06 (7.6·10^−3^)
	**EUR**	-0.65 (-0.69..-0.61)	-2.04	-1.02	-0.77	-0.55	-0.25	0.70	0.18 (6.8·10^−5^)
	**AMR**	0.21 (0.15..0.26)	-1.24	-0.22	0.07	0.35	0.65	1.58	-0.56 (1.2·10^−29^)
	**SAS**	0.42 (0.38..0.46)	-0.80	0.08	0.31	0.50	0.77	1.76	-0.29 (6.1·10^−11^)
	**EAS**	1.27 (1.24..1.30)	-0.32	0.98	1.18	1.37	1.58	2.52	0.12 (8.1·10^−3^)
	**AFR**	1.57 (1.54..1.60)	0.28	1.24	1.46	1.68	1.94	2.83	-0.41 (2.2·10^−28^)

The order of PRS_CHD_ and PRS_T2D_ distributions follow the same pattern: Europeans, including Estonians, are getting lower scores than Americans and South-Asians on both plot. East-Asians and Africans are getting the highest scores. However, when looking at the means of the distributions, the vast shifts between the populations can easily be observed. For instance, the highest quintile of Europeans (people having the highest genetic risk of CHD) have values ranging from -0.28 to 0.82. At the same time, this is approximately the range where Africans have the lowest quintile (-0.45 to 1.39) and therefore should have a lower risk. A similar difference appears in T2D.

**Fig 3:**
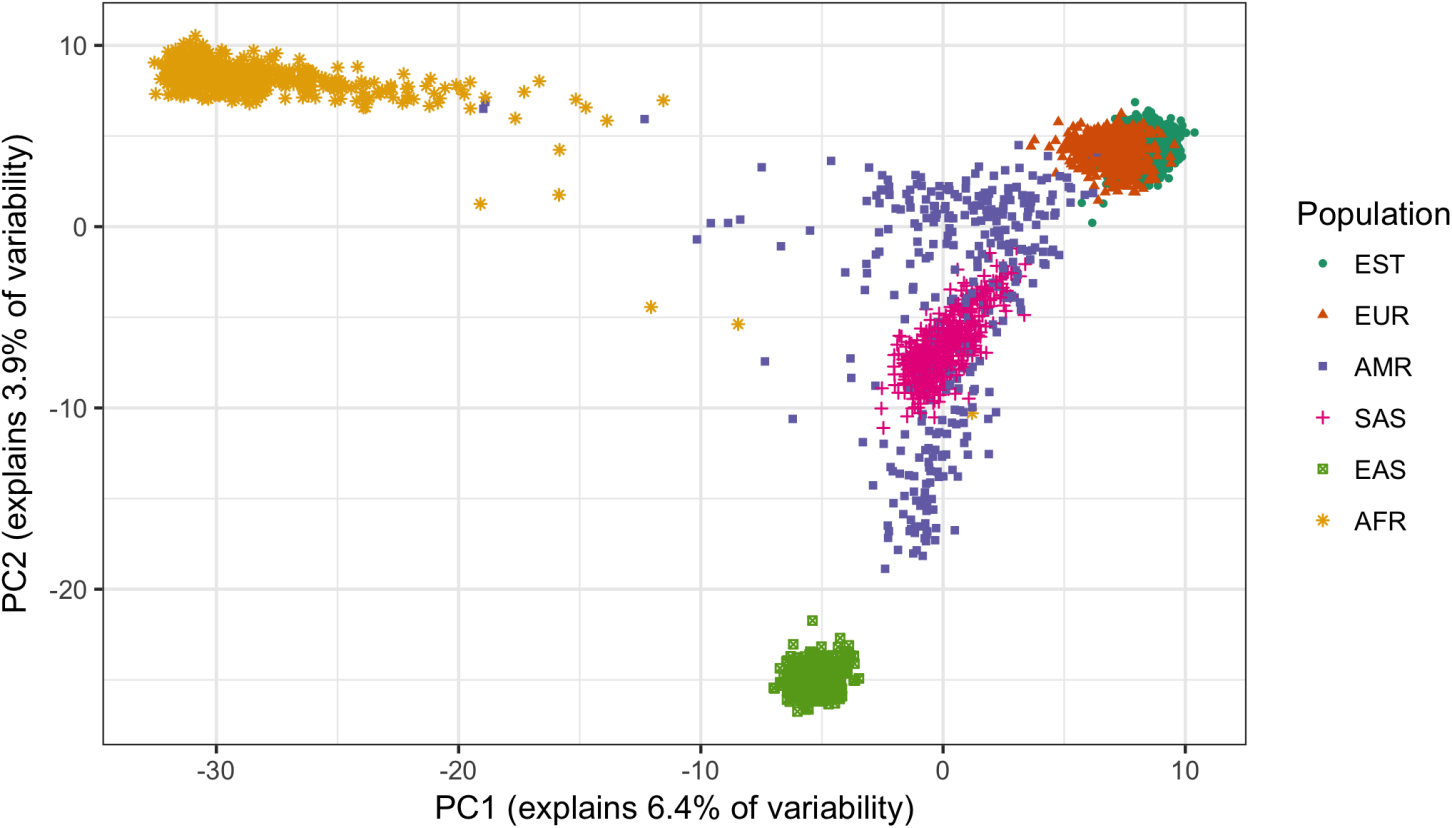
PCA plot of the samples, based on 7395 SNPs from PRS_T2D_, indicates that SNP data is population-specific.

PCA plot of all samples, based on 7395 SNPs from T2D model, is shown in Fig 3. PCA plot for CHD model is almost identical (available in S1 Fig).

For each population, the correlation between the first component of PCA, conducted only on the SNP data of that population, and PRS is given in Table 1. The correlation is very strong within American and African populations.

**Fig 4:**
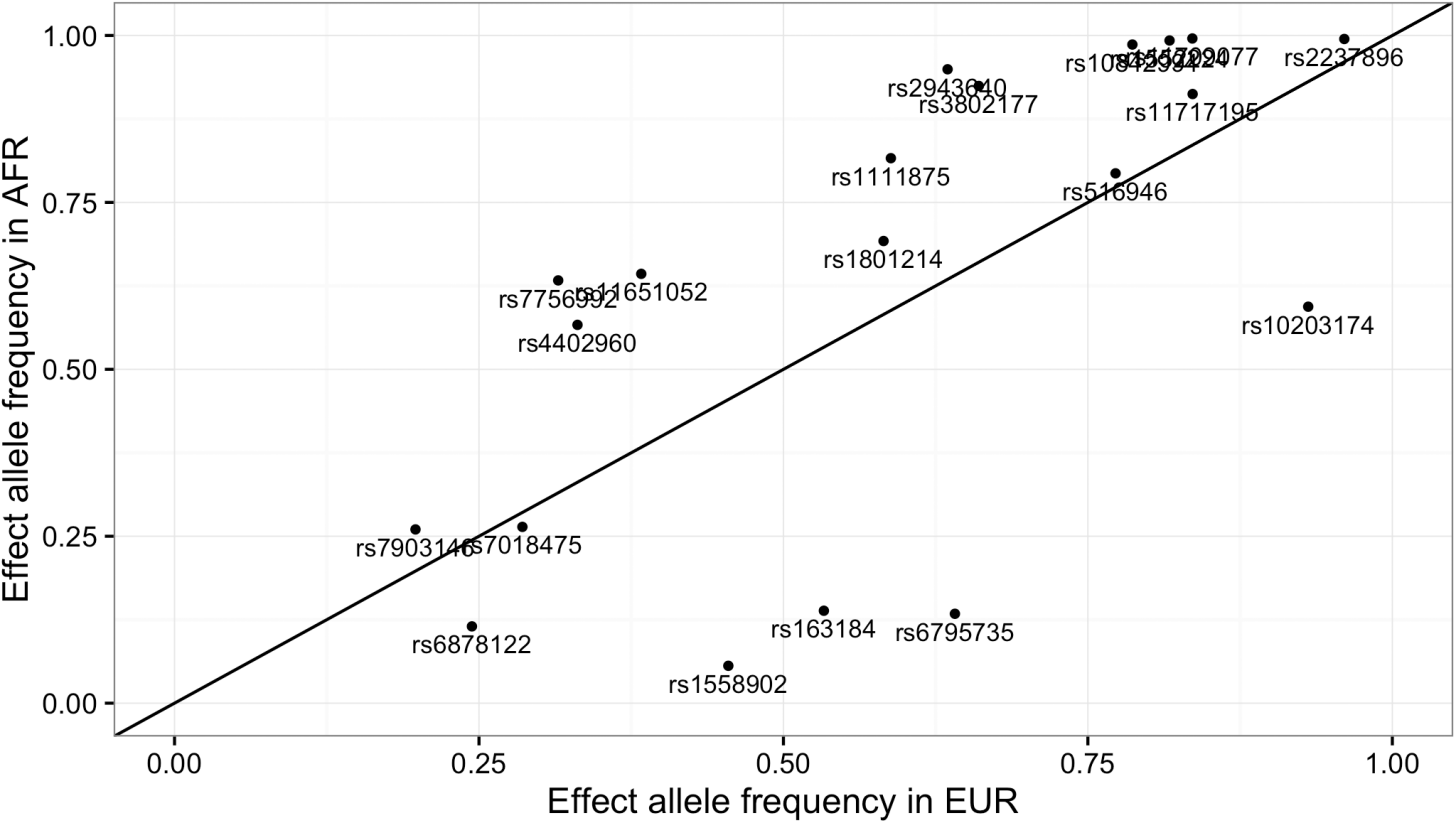
Comparison of effect allele frequencies of 20 top SNPs from T2D model in European and African population.

In order to illustrate the differences in effect allele frequencies of SNPs that have the strongest effect in the model, we have taken 20 top SNPs from the T2D model and compared their effect allele frequencies in African and European population in Fig 4. In this figure, effect allele is the allele which increases the risk score (has positive weight). It can be observed that frequencies tend to be higher in African than European population, which considerably is the cause of getting higher PRS values.

## Discussion

We calculated two polygenic risk scores PRS_CHD_ and PRS_T2D_, both containing large number of SNPs, for the samples from different populations and compared their distributions. We found that the distribution plots for both PRS models follow a similar pattern–the distribution parameters are considerably different. Estonians, together with other Europeans, tend to get the lowest and Africans the highest scores among considered demographic groups. Large shifts mean that the absolute ranges of the quintiles can be very different in different populations. The absolute score which in one population indicates the highest risk may mean the lowest risk in the other. If we apply the genetic risk cut-offs from European ancestry in individuals of African ancestry, then everyone would have an extremely high estimated risk level. Although the prevalence of the disease in different populations is indeed slightly different [20], it does not explain such a large variability in PRS distributions and stratifying the entire population to a high risk group does not make sense.

One might argue that the importance of absolute cut-off values is questionable because usually relative PRS thresholds are used in research instead. This holds only for research domain, where one is mainly interested in the strength of PRS-phenotype association and the absolute values are not important. However, if a PRS is used for personalised risk prediction in clinical practice, absolute thresholds are needed. Therefore, these PRS models together with their absolute thresholds, which were designed to the data of European populations, cannot be applied directly to other populations for risk estimation.

These findings are coherent with Martin et al. [13] who repeated PRS calculations for different models up to several hundred SNPs. They also found that in different populations the distributions vary. We can see that by using thousands of SNPs, the distribution plots of large PRS models are more likely to drift apart.

Carlson et al. provide an explanation for observing higher scores for non-European populations. They argue that because of the linkage disequilibrium in GWAS studies which are conducted mainly on European ancestries, discovered rare disease-associated variants are often not the true causal variants. As the linkage disequilibrium between causal and associated SNPs varies in different populations, the effect size of the disease-associated variant tends to be over-estimated in non-European ancestries for approximately a quarter of SNPs [15]. We also observed that most contributing (largest z-score) SNPs in our models tend to have higher effect allele frequencies in African populations compared to Europeans (Fig 4), consequently leading to relatively higher scores.

As a result of different effect allele frequencies, SNP data that is used for PRS calculation already includes the population information. The PCA plot in Fig 3 confirms that the populations are different when viewed from the 7395- (Fig 3) or the 45996-SNP perspective. That is, even before applying any weighting in the PRS model, populations already differ considerably from each other, making the starting point of using the model unequal. It is coherent with Lu et al. [21] who used 7775 SNPs (different from our models) from an older release of 1000G data for PCA plotting and found that African, European, and Asian ancestries are clearly distinguishable from each other, while the American population is admixed.

In order to overcome the PRS distribution shift problem the final score or SNP weights individually have to be adjusted according to the particular population where the score is applied.

So far, the large-scale GWAS studies have found relatively little between-cohort heterogeneity in the effects of individual SNPs. We cannot distinguish, whether it is so because there is not enough power to detect that or whether the effect sizes are actually homogenous–research so far supports the latter. Thus, there is no evidence to support differential weighting of individual SNPs. As the differences in PRS distribution depend mainly on different allele frequencies across populations, it seems justified to apply a population-specific correction to the entire PRS (rather than individual SNPs), to make the correct decision on general genetic risk level of any given individual.

One option is to simply recalculate the PRS distribution cut-offs for given target population by using sample data from the same population as a reference. This would solve the problem relatively easily for homogeneous populations. However, the problem still arises in admixed populations, where an individual might have a mixed set of SNPs from several ancestries and his/her individual cut-off thresholds do not match with the others. In such cases, first, we have to detect all these ancestries and then apply corresponding score adjustments to these populations.

However, even in a relatively homogeneous population or discovery-cohort, there is a potential risk of misclassifying an individual into a wrong population which would lead systematically to a wrong risk estimation. It can be observed from Table 1 that there is a significant correlation between the PRS and the first component of PCA analysis, especially for Africans and Americans. Due to the correlation, a person who is misclassified to a wrong population, will also get extreme PRS values and as risk score quintiles differ in different populations, this will lead to wrong risk estimation. This highlights the importance of correct population detection. Even in the discovery-population, in order to apply personalised medicine approaches like PRS-based risk estimation for an individual, he should always be tested beforehand to verify his descent from the same population.

How to detect the true mixture of ancestries for an individual effectively and taking it into account when adjusting PRS, remain an open question. We are getting incomparable scores because of the differences of the effect allele frequencies between populations, and at the same time, in order to suppress these differences, we have to know the descent of the individual. That brings us back to the PCA plot where we saw that the sample already holds the information about the ancestry. Can the same information be used for adjusting the score in-place? For instance, by weighting SNPs accordingly, by using the characteristics of the sample. We believe this issue deserves further investigation.

Adjusting the weights according to the descent require a trans-ethnic understanding of the disease-associated SNPs. Gathering such information is a tremendous challenge and this might be also the reason, why we did not find any such PRS models during the writing of this paper that has at least thousand SNPs and is built on global GWAS data.

## Supporting information

S1 File List of missing SNPs that were left out from analysis. (XLSX)

S2 File PLINK output files. (ZIP)

S1 Fig PCA plot of the samples, based on 45996 SNPs from PRS_CHD_. (TIF)
