# Supplementary figures and images for: Comparing distributions of polygenic risk scores of type 2 diabetes and coronary heart disease within different populations
Source: PLoS One. 2017 Jul 5;12(7):e0179238. doi: 10.1371/journal.pone.0179238 (PMC5497939; doi:10.1371/journal.pone.0179238)

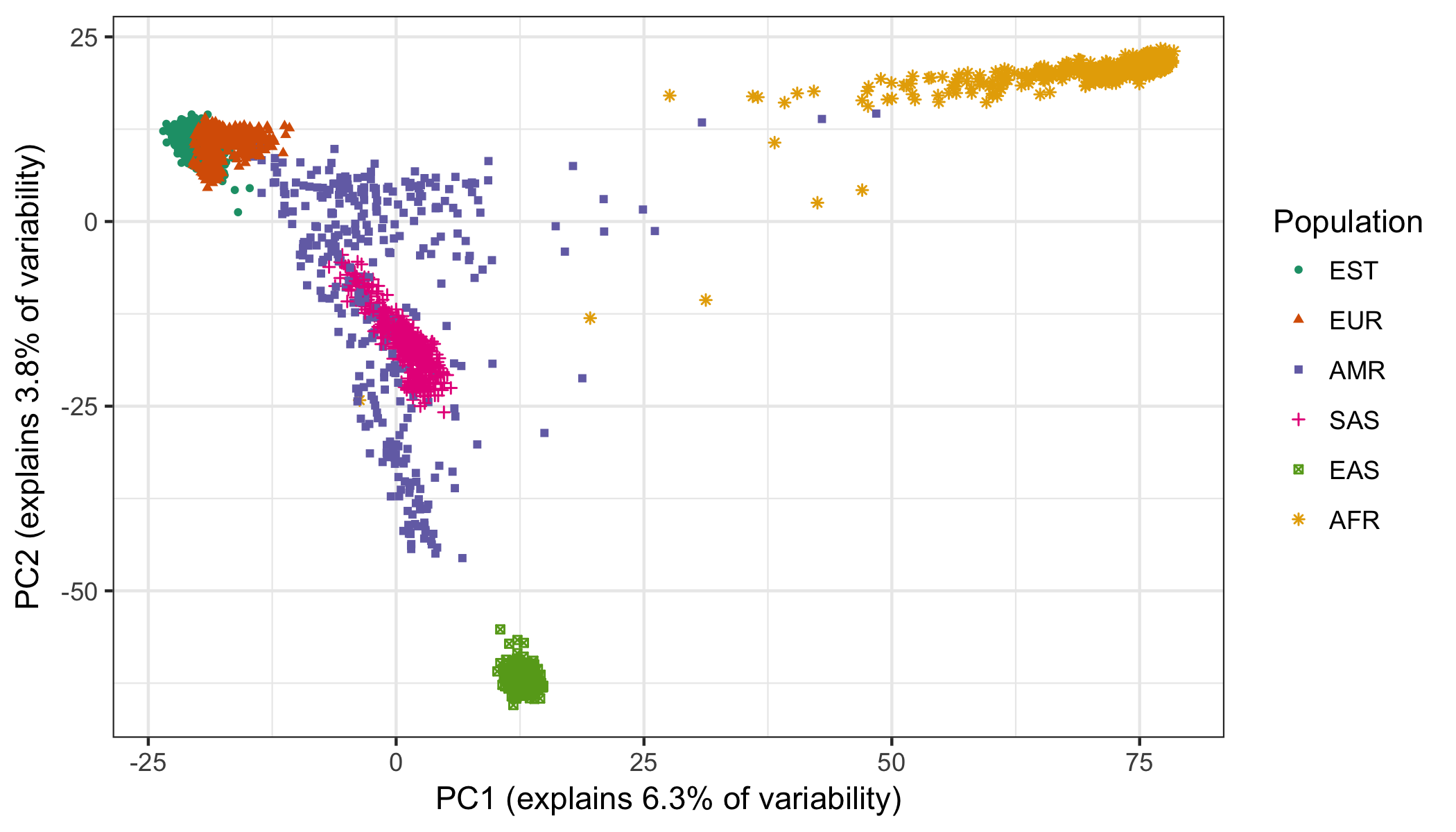

Supplement: S1 Fig — (TIF) [file pone.0179238.s003.tif]
